# Antibiotic Resistance in Bacterial Pathogens

**DOI:** 10.3390/antibiotics12030451

**Published:** 2023-02-24

**Authors:** Sara M. Soto

**Affiliations:** ISGlobal, Hospital Clínic-Universitat de Barcelona, Global Viral and Bacterial Infections Programme, 08036 Barcelona, Spain; sara.soto@isglobal.org

The increasing number of infections caused by antibiotic-resistant bacterial pathogens over the last few decades has become a critical global health problem, the scale of which has led to it being named a “silent pandemic”. For common bacterial infections, high rates of resistance against the antibiotics frequently used to treat them have been observed worldwide, indicating that we are running out of effective antibiotics. For ex-ample, resistance rate to ciprofloxacin, an antibiotic commonly used to treat urinary tract infections (UTIs), varied from 8.4% to 92.9% for Escherichia coli in countries reporting to the Global Antimicrobial Resistance and Use Surveillance System (GLASS). If no measures are taken, there will be an estimated 10 million deaths caused by resistant bacteria before 2050, 2 million more than those caused by cancer. Antibiotics are not only used to treat bacterial infections; they are also prescribed as prophylactics to patients undergoing various procedures, from joint replacements to chemotherapy. Therefore, the spread of antimicrobial resistance (AMR) will have crippling effects and severe patient costs, being much farther-reaching than those incurred from infection treatment only.

The research community is focusing many efforts on the discovery of new antibiotics, the study of the mechanisms of resistance, epidemiology studies, the development of new tools for a rapid diagnostic of resistant bacteria, the study of the spread of AMR, new ways to limited it, etc.

In this Special Issue, 14 papers on AMR have been published representing different areas of study in this field. 

Most studies of AMR acquisition are carried out using “in vitro” experiments. In this Special Issue, evidence for the “in vivo” development of resistance in *Salmonella* due to treatment-associated selection is reported [1]. Another important aspect of AMR is the study of the evolution of resistance. Thus, an increase in the percentage of vancomycin-resistant *S. aureus* [2], the prevalence of ESBL CTX-M-15 [3], and the different resistance patterns among species [4,5,6] support the idea that the knowledge of local distribution and susceptibility profiles of bacterial pathogens is essential for adequate clinical management.

Another important point of interest in the area of AMR is the spread of resistance by mobile genetic elements, such as plasmids, that can be exchanged between strains from the same or different species [7] and among strains from different niches (human, animal, food, and the environment) [8]. For this reason, to have an One Health vision is very important. 

In terms of diagnosis, rapid tools focused on lateral flow systems are being developed, especially for betalactamases [9] and carbapenemases [10] detection.

Finally, the development of new therapies, from antibiotic combinations [11,12,13] to the development of new antibiotics [14], is needed to combat AMR. 

All these areas are covered in this issue, which has been organized in honor of Prof. Jordi Vila, one of the most important researchers in the area of AMR.
